# Antenna proton sensitivity determines photosynthetic light harvesting strategy

**DOI:** 10.1093/jxb/ery240

**Published:** 2018-06-28

**Authors:** Eliška Kuthanová Trsková, Erica Belgio, Anna M Yeates, Roman Sobotka, Alexander V Ruban, Radek Kaňa

**Affiliations:** 1 Institute of Microbiology, Academy of Sciences of the Czech Republic, Opatovický mlýn, Třeboň, Czech Republic; 2 University of South Bohemia in České Budějovice, Faculty of Science, Branišovská, České Budějovice, Czech republic; 3 School of Biological and Chemical Sciences, Queen Mary University of London, London, UK

**Keywords:** *Chromera velia*, *in vitro* quenching, light harvesting strategy, non-photochemical quenching, NPQ kinetics, photoprotection, quenching p*K*_a_, violaxanthin

## Abstract

Photoprotective non-photochemical quenching (NPQ) represents an effective way to dissipate the light energy absorbed in excess by most phototrophs. It is often claimed that NPQ formation/relaxation *kinetics* are determined by xanthophyll composition. We, however, found that, for the alveolate alga *Chromera velia*, this is not the case. In the present paper, we investigated the reasons for the constitutive high rate of quenching displayed by the alga by comparing its light harvesting strategies with those of a model phototroph, the land plant *Spinacia oleracea*. Experimental results and *in silico* studies support the idea that fast quenching is due not to xanthophylls, but to intrinsic properties of the *Chromera* light harvesting complex (CLH) protein, related to amino acid composition and protein folding. The p*K*_a_ for CLH quenching was shifted by 0.5 units to a higher pH compared with higher plant antennas (light harvesting complex II; LHCII). We conclude that, whilst higher plant LHCIIs are better suited for light harvesting, CLHs are ‘natural quenchers’ ready to switch into a dissipative state. We propose that organisms with antenna proteins intrinsically more sensitive to protons, such as *C. velia*, carry a relatively high concentration of violaxanthin to improve their light harvesting. In contrast, higher plants need less violaxanthin per chlorophyll because LHCII proteins are more efficient light harvesters and instead require co-factors such as zeaxanthin and PsbS to accelerate and enhance quenching.

## Teaser

The extent of proton sensitivity in antenna proteins determines the photosynthetic light harvesting strategy in plants and algae.

## Introduction

Although under low light more than 83% of absorbed photons can be converted into chemical energy (e.g. Jennings *et al.*, 2005; Wientjes *et al.*, 2013), prolonged high light exposure rapidly switches photosystems to energy-dissipating states that release excess energy as heat (Demmig-Adams, 1990; Kaňa and Vass, 2008; Ruban *et al.*, 2012). The switch from light-harvesting to energy-dissipation mode has long been investigated, resulting in various models for various autotrophs (Demmig-Adams, 1990; Horton *et al.*, 1996; Kaňa *et al.*, 2012; Pinnola *et al.*, 2013; Erickson *et al.*, 2015; Büchel, 2015).

In higher plants, several processes contribute to excess light energy dissipation, but only the pH-dependent one, the so-called energy-dependent quenching mechanism (non-photochemical quenching; NPQ) is considered photoprotective (Demmig-Adams and Adams, 1992; Ruban, 2013). Proof of a strict connection between NPQ and pH is that reverse ATPase activity can stimulate NPQ even in the dark (Gilmore and Yamamoto, 1992). Besides controlling xanthophyll cycle activity, in several phototrophs pH exerts a direct control on NPQ. This is thought to act via a regulation of antennas (e.g. Dekker and Boekema, 2005; Horton *et al.*, 2008; Peers *et al.*, 2009; Grossman *et al.*, 2010). Indeed, a very similar thermal dissipation process to that *in vivo* can be induced *in vitro* in purified antennas by lowering pH and detergent concentration (Ruban *et al.*, 1994*a*). Starting from this evidence, it was proposed that antenna aggregation is at the basis of the NPQ process (Horton *et al*, 1996), and subsequent findings employing liposomes started to clarify how pH and ions together with lipids and lipid to antenna ratios control the ‘aggregation state’ of antennas (Moya *et al.*, 2001; Kirchhoff *et al.*, 2008; Akhtar *et al.*, 2015; Kaňa and Govindjee, 2016; Natali *et al.*, 2016; Crisafi and Pandit, 2017). In higher plants, antennas are the site of energy dissipation, whilst xanthophylls and the PsbS protein seem to be simply controllers of the process (Noctor *et al.*, 1991; Walters *et al.*, 1994; Li *et al.*, 2000; Betterle *et al.*, 2009). Evidence that *npq1*, a mutant lacking zeaxanthin, and *npq4*, a mutant without PsbS, could both perform NPQ indicated their dispensability, thus placing antennas and pH as the *only* key elements of the process (Niyogi, 1999; Johnson *et al.*, 2009; Johnson *et al.*, 2011). Nevertheless, xanthophylls play an important role modulating the kinetics of NPQ activation and dissipation (Johnson *et al.*, 2010). Pre-conditioning of leaves with light exposure, for instance, makes NPQ fast and persistent because of the conversion of violaxanthin into zeaxanthin (Ruban and Horton, 1999). Zeaxanthin, a highly hydrophobic pigment, in turn, makes antennas more dehydrated and therefore sensitive to pH and prone to quench compared with violaxanthin-enriched antennas. Interestingly, this idea was put forward not only for higher plant antennas (Ruban *et al.*, 1994*a*), but also for antennas from distant organisms such as diatoms (Gundermann and Büchel, 2008), brown algae (Ocampo-Alvarez *et al.*, 2013) and alveolates (Kaňa *et al.*, 2016).

The state-of-the-art model of NPQ for plants claims that, under high light, lumen acidification induces antenna protonation, which in turn triggers protein conformational changes, aggregation and energy dissipation. However, it seems that pre-aggregation *in vivo* can affect efficiency of antenna protonation and *vice versa* (e.g. Petrou *et al.*, 2014). Optical changes induced by aggregation can be visualized spectroscopically (Lokstein *et al.*, 2002), specifically as an increase in the fluorescence yield of red-shifted emission from antennas at low temperatures (Ruban *et al.*, 1991; Bassi and Dainese, 1992; Miloslavina *et al.*, 2008; Belgio *et al.*, 2012). Based on dicyclohexylcarbodiimide (DCCD) binding and mutagenesis work (Ruban *et al.*, 1998; Belgio *et al.*, 2013; Ballottari *et al.*, 2016), it was concluded that sensors for low pH are negatively charged residues located in a lumen-exposed antenna protein loop and in the C-terminus. Once protonated, those residues become neutral, thus making the whole protein more hydrophobic and easier to aggregate and quench. Although *in vitro* fluorescence quenching as a function of pH has been observed for various types of antennas (Gundermann and Büchel, 2012; Kaňa *et al.*, 2012; Schaller-Laudel *et al.*, 2015), identification of putative protonable residues so far concerned mainly antennas from the green lineage (Ruban *et al.*, 1998; Li *et al.*, 2004; Liguori *et al.*, 2013; Belgio *et al.*, 2013; Ballottari *et al.*, 2016).

Despite the progress in our understanding of NPQ in higher plants, this subject has been less explored in algae. The alveolate *Chromera velia* represents an interesting system in this context, as it shows efficient non-photochemical quenching (Kotabová *et al.*, 2011; Quigg *et al.*, 2012; Mann *et al.*, 2014) with similarities on the one side to higher plants, and on the other to brown algae and diatoms (see below). Isolated from stony corals from Sidney harbor, this facultative symbiont is globally distributed in the marine environment at depths not exceeding 5 m (Obornik and Lukes, 2013). The phylogenic origin of the alga is complex. *C. velia* is an alveolate, and therefore closely related to dinoflagellates and other algae in the SAR clade (such as diatoms and brown algae), but all phylogenic analyses have invariably demonstrated its genuine relationship to apicomplexan parasites (Oborník *et al.*, 2016). In any case, *C. velia* is considered a ‘red-clade’ alga, i.e. an alga whose chloroplast was obtained by secondary endosymbiosis from a red algal ancestor (Kotabová *et al.*, 2011; Sobotka *et al.*, 2017). In *C. velia*, NPQ is connected to the xanthophyll cycle (Kotabová *et al.*, 2011) as in brown algae (Ocampo-Alvarez *et al.*, 2013); however, differing from them (Garcia-Mendoza *et al.*, 2011) but similar to diatoms (Ruban *et al.*, 2004; Lavaud and Kroth, 2006; Grouneva *et al.*, 2008), its activation is extremely fast (almost monophasic) and pH-dependent (see Belgio *et al.*, 2018).

In the present paper, we investigated the reasons for the characteristic high rate of quenching displayed by the alga. We compared the NPQ of *C. velia* with that of a higher plant (a well-known system) and showed that the mechanism of heat dissipation and in particular NPQ *activation* is different in the two evolutionarily distant phototrophs. Our data indicated that the *Chromera* light harvesting complex (CLH) is more sensitive to protons than the higher plant antenna (light harvesting complex II; LHCII). We propose that protonation of the antenna is the basis of the ‘constitutively’ fast NPQ found in *C. velia* and, as previously suggested for diatoms (Lavaud and Kroth, 2006; Lavaud and Lepetit, 2013), ΔpH *by itself* is important for NPQ activation. This conclusion might also explain the unusual high light acclimation strategy recently reported for *C. velia*, consisting of a decrease in reaction centers whilst still maintaining a full antenna content (Belgio *et al.*, 2018).

## Materials and methods

### Plant material

*Chromera velia* (strain RM12) was grown in artificial sea water with additional f/2 nutrients (Guillard and Ryther, 1962). Cells were cultivated in glass tubes at 28 °C, in a continuous light regime of 200 µmol m^−2^ s^−1^ while aerated with air.

*Spinacia oleracia* (spinach) was purchased from a local supermarket. Intact chloroplasts were prepared as previously described (Crouchman *et al.*, 2006).

### Isolation of *C. velia* and plant light harvesting complexes

*C. velia* cells were broken and solubilized as described in Kaňa *et al.* (2016) and then loaded on a fresh, continuous 5–15% sucrose density gradient prepared using a home-made gradient maker in buffer containing 25 mM HEPES pH 7.8 and 0.04% *n*-dodecyl β-D-maltoside (β-DM). The ultracentrifugation was performed at 140 000 *g* at 4 °C for 20 h (with rotor SW28, for 40 ml tubes, of an L8-M ultracentrifuge; Beckmann, USA). The resulting band no. 2 contained a strong double band at 18 and 19 kDa, previously identified as ‘fucoxanthin chlorophyll *a*/*c* binding protein (FCP)-like antenna’ (Tichy *et al.*, 2013). The band analysis by Pan *et al.*, (2012) and Tichy *et al.* (2013) placed this antenna protein within the main FCP-like group of light-harvesting complexes and so it was named *Chromera* light harvesting complex (CLH).

After separation by sucrose gradient, the antenna protein was desalted using a PD10 column (GE Healthcare) in a buffer containing 20 mM HEPES (pH 7.6) and 0.01% (w/v) β-DM. Spinach LHCIIb was isolated as previously described (Ruban *et al.*, 1994*b*) and then purified, desalted and eluted in the same buffer as CLH. In both cases, antennas were isolated from samples dark-adapted for 30–45 min.

### Non-photochemical fluorescence quenching in native cells and isolated chloroplasts

Chlorophyll fluorescence was measured using a double modulation fluorometer FL-3000 (Photon System Instruments, Czech Republic). A multiple turnover saturating flash was applied to measure the maximum quantum yield of the photochemistry of photosystem II (*F*_v_/*F*_m_) according to (*F*_m_−*F*_0_)/*F*_m_, where the difference between the maximum (*F*_m_) and minimum (*F*_0_) fluorescence is used to calculate the variable fluorescence (*F*_v_) (van Kooten and Snel, 1990). Cells were then illuminated with an orange actinic light (625 nm, 500 µmol photons m^−2^ s^−1^), during which periodic saturating flashes were applied. NPQ was calculated as (*F*_m_−*F*_m_′)/*F*_m_ or *F*_m_′, where *F*_m_′ is the maximum fluorescence measured in the presence of actinic light. Non-photochemical quenching of fluorescence was measured in whole cells of *C. velia* (chlorophyll concentration 0.7 µg ml^−1^) and isolated spinach chloroplasts (chlorophyll concentration 1.4 µg ml^−1^). NPQ formation rates (NPQ as a function of time) in different xanthophyll cycle de-epoxidation states (DEPSs) were determined from the measured fluorescence traces as described in the ‘Data analysis and model fitting’ section.

Where indicated (Fig. 2; Supplementary Fig. S1 at *JXB* online), the effect of an uncoupler on the fluorescence quenching was examined by adding NH_4_Cl (final concentration of 15 mM) at different time points of the measuring protocol.

### *In vitro* fluorescence quenching of antennas

Isolated antennas (OD_676_=1 cm^−1^), solubilized in 0.01% DM, were diluted 20 times, while constantly stirring, in a room temperature buffer containing 10 mM sodium citrate and 10 mM Tris–HCl and adjusted with small drops of HCl to give the desired pH (for further details see Ruban *et al.*, 1994*a*; Belgio *et al.*, 2013). Chlorophyll fluorescence was continuously monitored using an FL 3000 fluorometer (PSI, Czech Republic, blue excitation at 464 nm, 184 µmol m^−2^ s^−1^). The p*K*_a_ values for quenching kinetics were calculated as described in the ‘Data analysis and model fitting’ section.

### Absorption measurement

Absorption spectra were recorded with a Unicam UV 500 spectrometer (Thermo Spectronic, UK).

### Pigment extraction and HPLC analysis

Cells or chloroplasts were collected on GF/F filters (Whatman, UK) and soaked in 100% methanol (overnight at −20 °C) and disrupted using a mechanical tissue grinder. Filter and cell debris were removed by centrifugation (12 000 *g*, 15 min) and the supernatant used for absorbance measurements at 652, 665, and 730 nm. Chlorophyll concentration was determined according to Porra *et al.* (1989). HPLC was carried out on an Agilent 1200 chromatography system equipped with a diode array detector. Pigments were separated on a Luna Phenomenex C8 (2) column (particle size, 3 µm; pore size, 100 Å; dimensions, 100 × 4.6 mm), by applying a 0.028 M ammonium acetate–MeOH gradient (20/80) as described in (Kotabová *et al.*, 2011) and the eluted pigments were quantified at 440 nm. The de-epoxidation state of the xanthophyll cycle pigments (DEPS) was calculated as: (zeaxanthin+0.5 antheraxanthin)/(violaxanthin+antheraxanthin+zeaxanthin) (Johnson *et al.*, 2009; Kotabová *et al.*, 2011; Oborník *et al.*, 2011). For purified antennas, the same procedure was applied simply skipping the first step of filtration through GF/F filter.

### Zeaxanthin enrichment

Plant chloroplasts and *C. velia* cells with a DEPS of 10% were obtained from dim-light-adapted samples (30 min). Enrichment in zeaxanthin was achieved as described previously (Ruban *et al.*, 1994*b*; Belgio *et al.*, 2014) by pre-conditioning leaves with 350 µmol photons m^−2^ s^−1^ under 98% N_2_ for 20–40 min for 20% and 40% DEPS, respectively. For *C. velia*, 10 min illumination with 500 µmol photons m^−2^ s^−1^ was sufficient to obtain 40% DEPS, in agreement with what has been previously published (Kotabová *et al.*, 2011). DEPS was assessed by immediate incubation in methanol followed by HPLC analysis (see ‘Pigment extraction and HPLC analysis’ section).

### *In silico* studies

For *in silico* studies, the LHCIIb structure resolved at 2.5 Å resolution (PDB code: 2BHW; Standfuss *et al.*, 2005) was employed. The structure of the CLH polypeptide (CveliaI_19753.t1 taken from Tichy *et al.* (2013)) was predicted using Phyre^2^ (http://www.sbg.bio.ic.ac.uk/phyre2/html/page.cgi?id=index) and YASARA software (http://www.yasara.org). The protonation states of protein ionizable groups were computed in both cases using the H++ program (http://biophysics.cs.vt.edu), an automated system that calculates p*K* values of ionizable groups in macromolecules and adds missing hydrogen atoms according to the specified pH of the environment. Results shown for LHCIIb are relative to chain A, but results for chains B and C were very similar, in agreement with (Xiao *et al.*, 2012). As recommended for typical physiological conditions and deeply buried residues, the external dielectric value was set to 80, the internal dielectric value to 4, salinity to 0.15 and pH to 7.5.

### Data analysis and model fitting

**Fig. 1.**
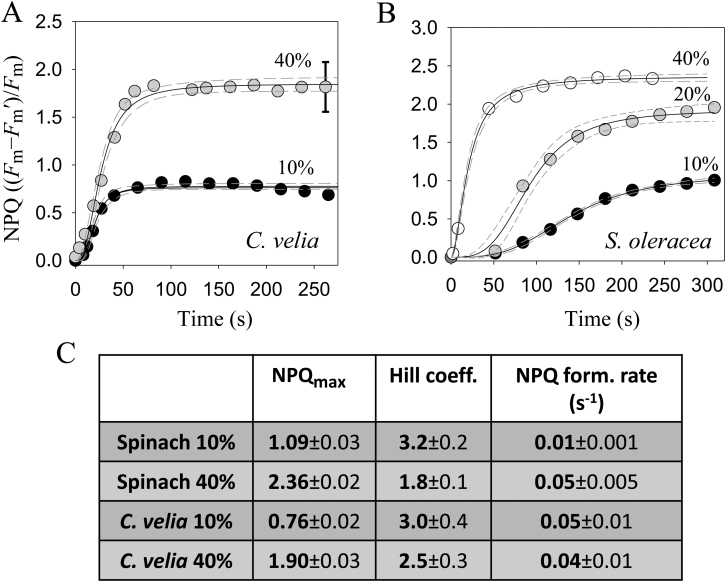
Fast NPQ formation rate in *C. velia.* Comparison between NPQ formation in *C. velia* cells (A) and intact spinach chloroplasts (B) in different de-epoxidation states (10, 20, or 40%). Circles, average data from three to six independent measures; solid lines, fittings; dashed lines, 95% confidence intervals. The error bar shows a typical standard deviation of the data. (C) Fitting parameters and relative errors obtained using the sigmoidal Hill equation *y*=[*ax*^*b*^]/[*c*^*b*^+*x*^*b*^], where *a* is NPQ_max_, *b* is the sigmoidicity parameter (Hill coefficient) and 1/*c* is NPQ formation rate (for more details, see ‘Materials and methods’).

NPQ formation rates (NPQ in function of time; see Fig. 1) were determined using a well-established methodology valid for both algae and vascular plants (see Serôdio and Lavaud (2011) concerning the applicability of the Hill equation to NPQ in algae). Briefly, average data from three to six independent measurements of *C. velia* cells and spinach chloroplasts in different DEPSs were fitted using the sigmoidal Hill equation three-parameter implementation in SigmaPlot 12.5 (Systat Software, Inc., San Jose, CA, USA). The standard error of the estimate was between 0.02 and 0.08, meaning that ~95% of the data fell within 2% of the fitted line; moreover *R*^2^ values were above 0.97, thus confirming the appropriateness of the approach.

**Fig. 2.**
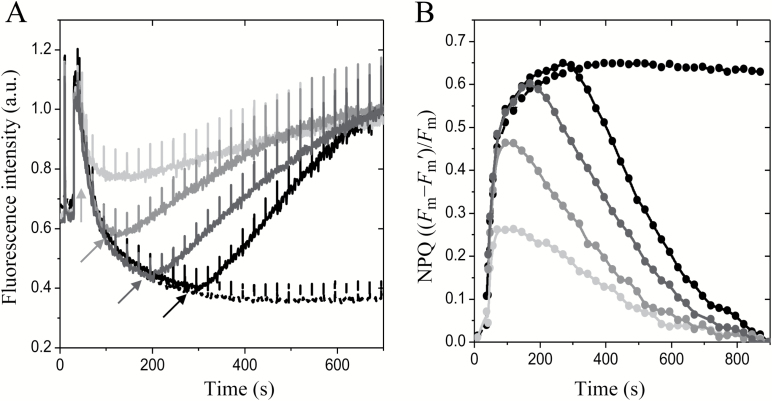
NPQ of *Chromera velia* cells is ∆pH-dependent. (A) Representative fluorescence induction traces showing the effect of the uncoupler NH_4_Cl on the NPQ of *C. velia* cells. Dashed black, control (no NH_4_Cl); light gray, NH_4_Cl added after 45 s; medium gray, uncoupler added after 99 s; dark gray, uncoupler added after 198 s; solid black, uncoupler added after 297 s. The actinic light intensity was 500 µmol m^−2^ s^−1^. Samples were dark adapted for 30 min before measurements. Care was taken to ensure that the sample was efficiently stirred throughout the whole experiment. For further information, see ‘Materials and methods’. (B) NPQ ((*F*_m_−*F*_m_′)/*F*_m_), calculated from the relative fluorescence traces shown in (A).

**Fig. 3.**
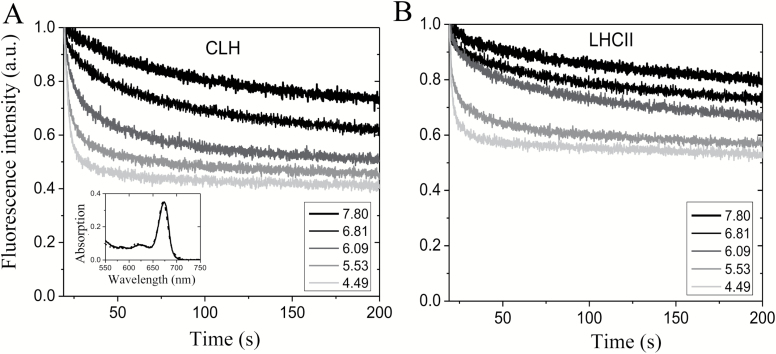
Quenching of *Chromera velia* antennas is highly sensitive to pH. Representative fluorescence time course of CLH (A) and LHCII (B) as a function of pH. Samples were injected into a buffer containing 0.0005% β-DM, 10 mM HEPES and 10 mM sodium citrate (final concentrations). Each buffer had been HCl-buffered to the pH indicated in the figure, prior to sample injection. Data were normalized to the fluorescence maximum. Inset: absorption spectrum of CLH at high (solid line, pH 7.8) and low (dashed line, pH 4.5) pH.

**Fig. 4.**
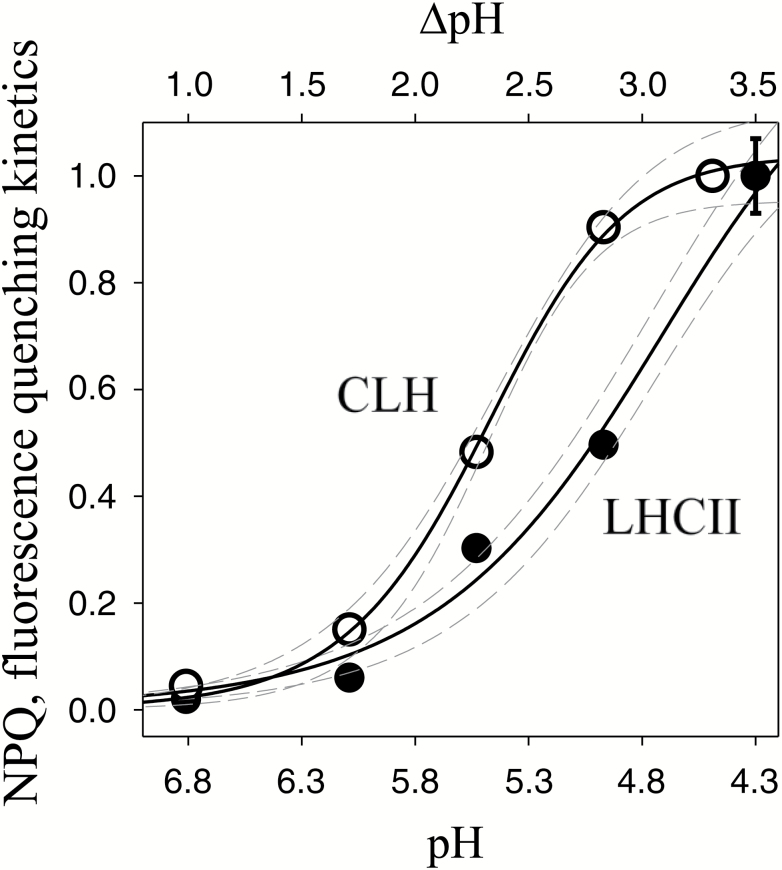
Comparison of pH titration curves for fluorescence quenching of LHCII and CLH. The relationship between percentage of quenching and pH was obtained from traces like those shown in Fig. 3 fitted as described in the ‘Data analysis and model fitting’ section of ‘Materials and methods’, using a previously established model (see e.g. Ruban and Horton 1999). Circles, mean averages from at least three independent replicates; solid lines, fittings; dashed lines, 95% confidence intervals. The error bar shows typical standard deviation.

In order to determine the quenching p*K*_a_ of antennas, we used a method previously established for various antennas including mutants (Ruban *et al.*, 1994*a*; Ruban and Horton, 1999; Belgio *et al.*, 2013; Zaks *et al.*, 2013). Briefly, the relationship between quenching kinetics and pH (see Fig. 4) and relative parameters (Table 1) were obtained from experiments like the one shown in Fig. 3 as follows. Quenching kinetics were calculated at each pH point by fitting the measured traces (Fig. 3) with the three-parameter hyperbolic decay function: *y*=(*y*_0_+*ab*)/(*b*+*x*) where 1/*b* represents the *rate* of the process. Then the data points from Fig. 4 were fitted by the sigmoidal Hill equation *y*=[*ax*^*b*^]/[*c*^*b*^+*x*^*b*^] in order to obtain Hill coefficients (*b*), p*K*_a_ values (*c*) and quenching kinetics at pH 4.97 (see also (Johnson *et al.*, 2012; Petrou *et al.*, 2014). The standard error of the estimate was again low (below 0.1) and *R*^2^ above 0.90, confirming the validity of the approach.

**Table 1.** pH versus quenching titration curve fitting parameters in CLH and LHCII

Sample	**Hill coefficient**	**Estimated p*K***_**a**_	**Quenching kinetics at pH 4.97**
LHCII	7.2 ± 1.9	5.0 ± 0.1	0.50 ± 0.01
CLH	7.5 ± 1.1	5.5 ± 0.1	0.90 ± 0.05

Titration parameters were determined by fitting measured traces like those represented in Fig. 3 as described in the ‘Data analysis and model fitting’ section of ‘Materials and methods’. Standard fitting errors were provided by SigmaPlot software. (For more details, see Ruban *et al.* 1994*a*; Ruban and Horton 1999; Petrou *et al.* 2014.)

## Results

The kinetics of non-photochemical quenching (NPQ) activation were studied for *C. velia* in different xanthophyll de-epoxidation states (DEPSs) and compared with that of *Spinacia oleracea* (spinach) (Fig. 1). NPQ formation rate positively correlated with the DEPS in spinach as 10, 20, and 40% de-epoxidation yielded significantly different NPQ formation rates of 0.006, 0.011, and 0.05 s^−1^, respectively (Fig. 1B), in agreement with current NPQ models and previous results from various phototrophs (see e.g. (Demmig-Adams, 1990; Ruban *et al.*, 1994*b*; Jahns and Holzwarth, 2012; Goss and Lepetit 2015). In contrast, NPQ in *C. velia* formed quickly regardless of the de-epoxidation state (Fig. 1A). Values between 0.04 and 0.05 s^−1^ were thus found for both 10 and 40% DEPS, showing fast NPQ formation, independent of xanthophyll content (Fig. 1C).

The lack of an evident kinetic effect of xanthophylls in *C. velia* can also be seen from the *shape* of the NPQ formation curve. Whilst in spinach the increase in zeaxanthin (zea) concentration from 10 to 40% reduced curve sigmoidicity from 3.2 to 1.8 (Fig. 1B; Table S1), in *C. velia* no evident change could be seen (Fig. 1A) and the Hill coefficients were not significantly different in the two conditions (3.0 ± 0.4 *versus* 2.5 ± 0.3; see Supplementary Table S1). The increased de-epoxidation (from 10% to 40%; Fig. 1A), therefore, did not seem to affect NPQ kinetics as strongly as in spinach, but it stimulated the total NPQ (NPQ_max_; see Table S1). This is in agreement with a previous report (Kotabová *et al.*, 2011) showing NPQ enhancement by zeaxanthin in *C. velia*.

In *C. velia*, NPQ was induced almost instantaneously with the turning on of the actinic light, and we therefore used NH_4_Cl to investigate the possibility that lumen acidification was the basis of fast NPQ. As with spinach, in *C. velia* NH_4_Cl reversed fluorescence quenching independent of its addition time during irradiation (Fig. 2A), proving a strict link between protons and NPQ in *C. velia*. However, the kinetics of NPQ relaxation at different time points were very different from each other and from those of spinach (Supplementary Fig. S1A). Whilst in spinach NPQ relaxed almost immediately after NH_4_Cl injection, with 70% fluorescence recovery within 10 s, it took at least 500 s to achieve a similar recovery in *C. velia* (Fig. 2B). Interestingly, in *C. velia*, the later NH_4_Cl was added, the faster NPQ relaxed (Fig. 2B). This is in strict contrast to higher plants (Supplementary Fig. S1B), where faster relaxation kinetics were observed at the beginning of NPQ formation (see e.g. Fig. 3A in (Ruban *et al.*, 2004), suggesting a different sensitivity of NPQ to lumen acidification. The connection between NPQ and protons was further investigated *in vitro* using isolated antennas.

‘Fluorescence quenching titration’ is an efficient way to systematically study the pH dependency of quenching *in vitro*, by injecting purified antennas into buffers of increasingly acidic pH (Ruban *et al.*, 1994*b*; Wentworth *et al.*, 2000; Kaňa *et al.*, 2012; Belgio *et al.*, 2013). This method was employed to assess the hypothesis that faster NPQ activation (Fig. 1) related to antenna protonation, rather than to zeaxanthin content. Therefore LHCII and CLH complexes were isolated from dark-adapted material and the absence of zeaxanthin was confirmed by HPLC analysis (see Supplementary Table S1 at *JXB* online).

Upon injection, CLH displayed a progressive quenching proportional to the acidity of the buffer (Fig. 3A). Sample integrity was constantly monitored by absorption spectroscopy (Fig. 3, inset) and by reversibility of the quenching after detergent addition (data not shown). Besides the general similarity of the process, pointing to a fundamentally conserved quenching mechanism, the differences between the two types of sample are notable. At each pH value, fluorescence quenching was consistently higher in CLH compared with LHCII, with the biggest difference found around pH 6.0. From the traces in Fig. 3, a titration curve of quenching kinetics as a function of pH was constructed (Fig. 4). It shows that, to attain the same rate of fluorescence quenching, a lower pH is required in LHCII compared with CLH. In particular, almost 50% of maximum quenching rate was observed at pH 5.5 in CLH, whilst a pH of 5.0 was necessary to get the same quenching rate in spinach. Similarly, CLH showed almost the maximum quenching rate (90 ± 5%) at pH 4.97, whereas for LHCII it was only 50%. This was reflected in a shift by 0.5 pH unit to higher values in the calculated quenching p*K*_a_ of CLH compared with LHCII, i.e. from 5.5 ± 0.1 to 5.0 ± 0.1 (Table 1). The p*K*_a_ value for LHCII was in good agreement with that previously reported (see e.g. p*K*_a_=4.9 in Petrou *et al.* (2014)). The Hill coefficient for CLH was not significantly different from that of LHCII (7.2 ± 1.9 and 7.5 ± 1.1 for LHCII and CLH, respectively; Table 1) and in both cases they were higher than those for *in vivo* quenching (see Fig. 1), consistent with the absence of zeaxanthin (see Supplementary Table S1 and Discussion). In summary, the shift in quenching p*K*_a_ confirmed a higher proton sensitivity of CLH compared with LHCII, independent of xanthophylls.

**Fig. 5.**
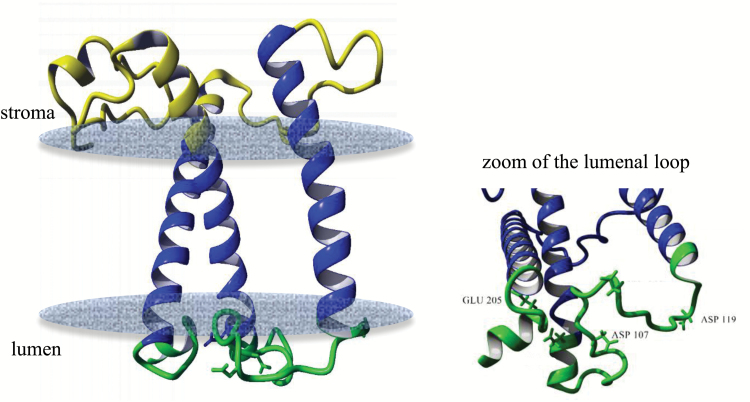
Predicted protein structure for CLH. Predicted structure of the CLH antenna based on sequence homology with LHCII. A zoom of the stranded lumenal loop is shown on the right. The putative residues involved in triggering NPQ are labelled in black. Blue, transmembrane helices; green, lumenal loop region; yellow, stromal loop. For the prediction of protein structure and the p*K*_a_ of residues, YASARA and H++ programs were used, respectively.

In order to address possible reasons behind the higher pH sensitivity found in CLH, a comparative *in silico* analysis was conducted using the amino acid sequences of CLH and LHCII (Supplementary Fig. S2). A schematic overview of the two proteins is presented in Supplementary Fig. S3. We have explored in particular the protein lumenal loop to identify residues that are protonable within the physiological range. The protein structure predicted for CLH is presented in Fig. 5. We found 24 negatively charged amino acidic residues in total (i.e. aspartic and glutamic acids) in CLH, four of which are located in the luminal loop (Glu-93, Asp-107, Asp-113, Asp-119) and one in the C-terminus (Glu-205).

The estimated *in situ* p*K*_a_ values were calculated and compared with LHCII (2.5 Å resolution structure from Standfuss *et al.* (2005)) and are presented in Table 2. Results for LHCII are in good agreement with a previous report (Xiao *et al.*, 2012), where two residues in particular (Glu-107 and Asp-215) were indicated as putative pH sensors for NPQ as their quenching p*K*_a_ values are within the thylakoid physiological range (3.9–7.5). The same analysis applied to CLH revealed the presence of three plausible protonable residues: Asp-107, Asp-119 and Glu-205 (see Table 2, Fig. 5 right). Furthermore, their p*K*_a_ values were shifted to higher pH values compared with LHCII, confirming that the lumenal loop is more sensitive to protonation in CLH (see Asp-107, Asp-119 and Glu-205 and their p*K*_a_ in Table 2).

**Table 2.** List of protonable lumenal loop residues (Glu, Asp) predicted by the H++ program for LHCII and CLH antenna proteins

LHCII		CLH	
Residue	p*K*_a_	**Residue**	p*K*_a_
Glu-94	1.5	Glu-93	1.0
**Glu-107**	**4.4**	**Asp-107**	5.9*
Asp-111	3.4	Asp-113	2.9
Glu-207	2.9	**Asp-119**	3.9*
Asp-211	3.5	Lys-211	7.4
**Asp-215**	**5.3**	**Glu-205**	>7*

The putative residues that can be protonated within the physiological pH range (3.9–7.5) are in shown bold. Residues in CLH with a higher p*K*_a_ than LHCII have been marked with an asterisk. Set values in the simulation were: for internal dielectric, 4; external dielectric, 80; and salinity, 0.15; in agreement with Xiao *et al.* (2012). Predicted sequences and protein structures are shown in Supplementary Figs S2 and S3, respectively.

An overall comparison between LHCII and CLH protein structures (Table 3) indicated that, despite a similar number of total protonable residues (~11.4–11.5% in both cases), CLH displayed a lower protein charge than LHCII at pH 7.6, that is −6 *versus* −24, respectively. This means that LHCII tends to be more charged than CLH and a stronger protein–protein repulsion is expected for LHCIIs at pH 7.6 (see Discussion). In agreement with this, the CLH isoelectric point was ~0.4 higher than LHCII, implying that ~30 times fewer protons are required to neutralize negatively charged residues compared with LHCII. In summary, the *in silico* results supported the experimental data well and provided theoretical explanations for the faster, more efficient quenching found for CLH.

## Discussion

In the present paper, we investigated reasons for fast NPQ activation in *C. velia*. In higher plants, the kinetics of NPQ induction are influenced by xanthophyll composition (Fig. 1B). Demmig-Adams (1990) was the first to provide evidence for a connection between the xanthophyll cycle and NPQ. She showed that the conversion of violaxanthin into zeaxanthin, stimulated under light by lumen acidification, strongly enhanced NPQ. Later it was noticed (Ruban and Horton, 1999) that the NPQ of zeaxanthin-enriched samples was much faster, as zeaxanthin changed the NPQ dependency (cooperativity) as a function of ∆pH, from sigmoidal (violaxanthin) to hyperbolic (zeaxanthin) (see also Horton *et al.*, 2000; Johnson *et al.*, 2009). Here, we confirmed with a control sample (spinach) that the transition into the quenched state is slower for leaves enriched in violaxanthin compared with zeaxanthin (Fig. 1B) as the Hill coefficient decreased in the presence of zeaxanthin. In *C. velia* however, we found a different behavior. Although NPQ was greater in zeaxanthin-enriched samples, confirming the first observations (Kotabová *et al.*, 2011), its *rate* was insensitive to xanthophyll composition (Fig. 1A, C), indicating that the reason for the fast NPQ in *C. velia* resided elsewhere. The NH_4_Cl-infiltration experiment (Fig. 2) following the procedure of Ruban *et al.* (2004) and Lavaud *et al.* (2002), suggested that fast NPQ related to lumen acidification and protons. Incomplete diffusion of the uncoupler was in fact ruled out by previous evidence of efficient NH_4_Cl penetration in *C. velia* cells (see Fig. 4b in Belgio *et al.*, 2018). The titration of NPQ as a function of pH confirmed that CLH was significantly more sensitive to protons than LHCII (Figs 2, 3). Most importantly, the *rate* of quenching was increased, especially between pH 5 and 6.5. Within this pH range a very rapid quenching formed almost instantly upon injection of a CLH sample (Fig. 3A). This resulted in a shift in quenching p*K*_a_ of CLH to higher pH values than LHCII (Table 1). Therefore, to attain 50% of maximum quenching kinetics, a 0.5 unit lower pH value (corresponding to 3.16 times more protons) was required for LHCII than CLH, indicating that the latter had an increased sensitivity to acidification. The high level of structural similarity between CLH and LHC protein families (Pan *et al.*, 2012; Tichy *et al.*, 2013; see also Supplementary Fig. S3) prompted an *in silico* comparison between lumenal loop residues. The analysis identified five protonable (i.e. negative) residues in the lumenal loop of CLH (Table 2). Three of them were predicted to be protonable within the physiological range (assuming a chloroplast lumen pH ranging between 3.9 and 7.5 (Peltier *et al.*, 2002). Compared with the corresponding residues in LHCII, the quenching p*K*_a_ values of these three residues are significantly higher in CLH (Table 2), which is consistent with the shift in the quenching p*K*_a_ found from experimental data (Fig. 4). We propose that during lumen acidification and *in vitro* quenching (Fig. 3), these residues shift from negative to neutral (i.e. become protonated) and this occurs earlier (i.e. at higher pH values) in CLH than in LHCII. This mechanism would explain the faster quenching (Fig. 3) and the shift to higher values of CLH quenching p*K*_a_ found experimentally (Fig. 4). Interestingly, the p*K*_a_ of Lys-211 (which in standard conditions has a p*K*_a_ of 10.67) was found to be reduced to 7.4 in CLH (Table 2). If confirmed by further studies, this means that this lumenal loop residue can also be protonated during lumen acidification and therefore might play a role in NPQ activation in *C. velia*. The comparison between LHCII and CLH (Table 3) indicated also that the algal antenna is less charged at physiological pH than LHCII. As protein clustering proved to be crucial for efficient quenching of LHCII (Betterle *et al.*, 2009; Johnson *et al.*, 2011; Belgio *et al.*, 2012; Petrou *et al.*, 2014), an overall less charged protein like CLH could be more prone to aggregate and therefore quench more easily, due to a minor protein–protein electrostatic repulsion. The predicted isoelectric point is consistent with this. For CLH, in fact, a ~0.4 lower p*I* was found (Table 3), corresponding to 30–40 less protons required for charge shielding. Considering the *in vitro* and *in silico* results together, we suggest that the increased NPQ formation kinetics relate to inbuilt antenna properties, in terms of both a higher number of lumen-exposed protonable residues and an overall increased protein hydrophobicity. We hypothesize that this applies also to other similar antennas, such as diatom FCPs. In fact, although CLH binds only chlorophyll *a* and xanthophylls (see Kotabová *et al.*, 2011), due to its structural properties, this protein was classified as ‘FCP-like’, i.e. closely related to antennas from dinoflagellates, brown algae, and diatoms (Lepetit *et al.*, 2010; Pan *et al.*, 2012; Tichy *et al.*, 2013). Moreover diatoms can also be characterized by fast NPQ activation (Ruban *et al.*, 2004; Lavaud and Kroth, 2006; Grouneva *et al.*, 2008).

**Table 3.** Comparison of total charges between LHCII and CLH

	**LHCII**	**CLH**
Protonable residues	25/218 (11.5%)	24/211 (11.4%)
p*I*	4.61	4.97
Charge at pH 7.6	−24	−6

Total number of protonable residues, the isoelectric point (p*I*) and the total protein charge of LHCII and CLH antenna proteins, as predicted by the H++ program. Relative sequences and protein structures are shown in Supplementary Figs S2 and S3, respectively.

**Fig. 6.**
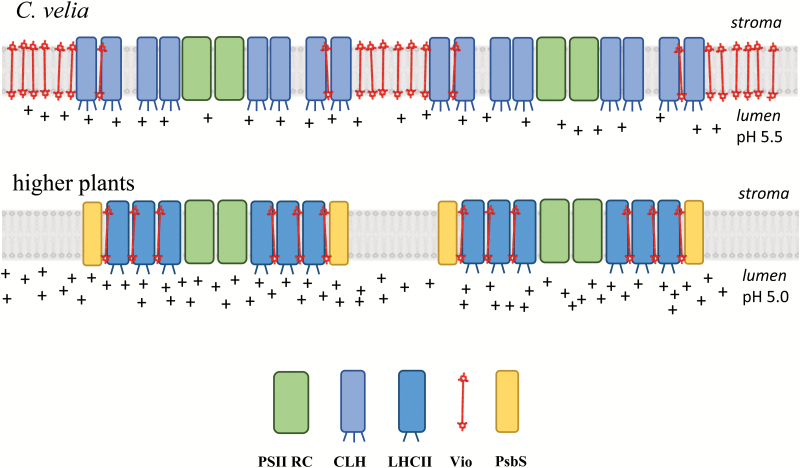
Scheme showing the different light harvesting strategies of *C. velia* and higher plants. The *C. velia* thylakoid membrane carries CLH proteins that are ‘natural quenchers’ with three protonable lumen-facing residues, D107, D119, and E205 (indicated by small protrusions). The membrane is highly enriched in unbound, ‘anti-quenching’, violaxanthin pigments, and PsbS protein is absent. The higher plant thylakoid membrane supports the LHCII protein, a ‘natural light harvester’ with two protonable lumen-facing residues. PsbS protein is required for effective quenching, and the amount of unbound violaxanthin in the membrane is negligible. The scheme does not represent real stoichiometries/proportions. For more details, see main text.

It was experimentally shown for brown algae (Nitschke *et al.*, 2012), alveolates (Belgio *et al.*, 2018), and other microalgae (Goss and Jakob, 2010) that the habitat and particularly the light conditions affect NPQ capabilities of algae from the SAR clade. As a coral symbiont, *C. velia* is expected to be mainly exposed to rather ‘moderate’ light intensities. However, as this organism can be also found ‘free-living’ outside the coral, at depths of 3–5 m, light intensities of up to 1000 μmol m^−2^ s^−1^ are normally experienced on a sunny day (Behrenfeld *et al.*, 1998; Oborník *et al.*, 2011). We can speculate that, due to fast quenching of antennas, in *C. velia* there was no selective pressure towards proteins capable of enhancing NPQ rate such as PsbS or Lhcsr (Pan *et al.*, 2012). These proteins in fact have a role as NPQ enhancers in vascular plants and green microalgae, respectively (Goss and Lepetit 2015). Spinach and *C. velia* seem therefore to have evolved very different ‘antenna behaviors’ in relation to different acclimation strategies. They can be summarized as follows (Fig. 6):

*Chromera velia* carries antenna proteins that are ‘natural quenchers’; PsbS, a strong NPQ enhancer (Li *et al.*, 2000), is absent (Pan *et al.*, 2011) and the thylakoid membrane is highly enriched in violaxanthin, an ‘anti-quenching’ pigment (Kaňa *et al.*, 2016). As a consequence its NPQ kinetics are characterized by fast formation/slow relaxation.
Higher plant antenna proteins, here represented by spinach LHCII, are ‘natural light harvesters’, so PsbS is required for effective but in particular *fast* quenching (Johnson and Ruban, 2011) and little or no free violaxanthin is present the membranes (Dall’Osto *et al.*, 2010; Xu *et al.*, 2015). The NPQ kinetics are characterized by slow formation/fast relaxation.

In this scenario, ‘free’ violaxanthin plays a role of quenching inhibitor, particularly important for *C. velia* and less crucial for LHCII. A similar role of violaxanthin was previously suggested for some brown algae (Ocampo-Alvarez *et al.*, 2013). It explains the abundance of violaxanthin in algae like *C. velia*, where the violaxanthin to Chl *a* ratio is ~0.36 (mol mol^−1^), ~8 times higher than in plants (see e.g. Kotabová *et al.*, 2011), which is supported by work showing quenching modulation by ‘free’, i.e. not firmly bound to protein, xanthophylls (Ruban *et al.*, 1994*a*; Lepetit *et al.*, 2010; Mann *et al.*, 2014; Xu *et al.*, 2015; Kaňa *et al.*, 2016).

Finally, the model presented (Fig. 6) provides an explanation also for the unusual acclimation strategy observed in *C. velia*: whilst plants (carrying ‘natural harvester’ antennas) protect themselves from high light by reducing their antenna size (see Kouřil *et al.*, 2013), in *C. velia* (characterized by ‘natural quencher’ antenna proteins) the antenna size is unaffected even after days of exposure to high light (Belgio *et al.*, 2018). This evidence, at first puzzling, seems now more logical in view of the results presented here.

## Conclusions

In conclusion, we have shown a similar quenching mechanism in antennas from a higher plant compared with those from an alveolate. In both cases the trigger is low pH and the likely sensors are protonable lumenal residues. However, the actual sensitivity to lowering pH is different for the two proteins as CLH is more sensitive to protons than LHCII. We propose that this is due to subtle differences in the amino acid composition of the protein lumenal loop. As a result, CLH switches into a dissipative quenched state more easily than LHCII and therefore the higher plant antenna protein can be considered a ‘natural light harvester’ whilst the CLH protein is a ‘natural quencher’.

## Supplementary data

Supplementary data are available at *JXB* online.

Fig. S1. NH_4_Cl induces fast NPQ relaxation in spinach chloroplasts.

Fig. S2. Sequence of LHCIIb and CLH used in the present study.

Fig. S3. Schematic overview of LHCII (left) and CLH (right) antenna protein structures used in the present study.

Table S1. Pigment composition of antennas isolated from *C. velia* and spinach.

Supplementary Table S1+Figures S1-S3

## Author contributions

RK, EKT and EB conceived the project; EKT and EB performed the experiments and analyzed the data; AMY provided HPLC technical assistance; RS provided experimental advice concerning protein isolation; EB, RK and EKT wrote the article with contributions from all the co-authors; EB, EKT and AMY prepared the Figures. AVR supervised and complemented the work.
