# Supplementary material for: Antenna proton sensitivity determines photosynthetic light harvesting strategy
Source: J Exp Bot. 2018 Jun 28;69(18):4483–93. doi: 10.1093/jxb/ery240 (PMC6093471; doi:10.1093/jxb/ery240)
Supplement: Supplementary Table S1+Figures S1-S3 [file ery240_suppl_supplementary_table_s1_figures-s1-s3.pdf]

Supplemental file

Antenna proton sensitivity determines photosynthetic light harvesting strategy

Eliška Kuthanová Trsková, Erica Belgio, Anna M. Yeates, Roman Sobotka, Alexander V. Ruban, and Radek Kaňa

	Neo	Vio	Ant	Lut (LHCII) Isofuco(CLH)	Zea	DEPs	xan/ chl
**LHCIIb**	29±1	4±1	0	67±1	0	0	0.35
**CLH**	0	30±1	0	69±3	0	0	0.9

Table S1
Pigment composition of isolated LHCIIb and CLH. Neo, Vio, Ant, Lut, Isofuco, Zea, DEPs: neoxanthin, violaxanthin, antheraxanthin, lutein, isofucoxanthin, zeaxanthin, de-epoxidation state (Z + 0.5A)/(V+A+Z) and xanthophyll/chlorophyll molar ratios. Data are presented as (xanthophyll/total xanthophyll)% and are means ± SD from 3 replicates, xan/chl: xanthophyll/chlorophyll molar ratio.

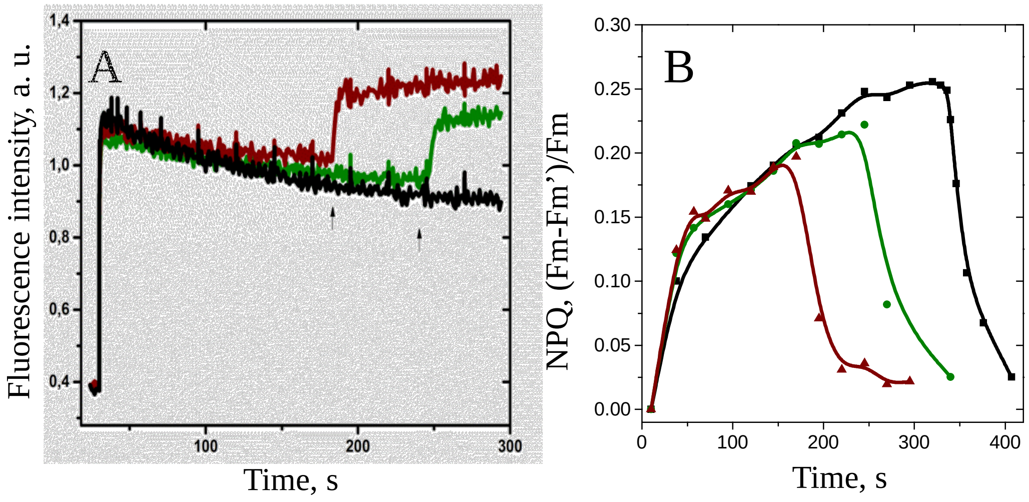

Figure S1. NH_4Cl induces fast NPQ relaxation in spinach chloroplasts. Panel A. Representative fluorescence traces showing the uncoupler-dependent reversibility of NPQ in intact chloroplasts isolated from spinach. Black, control (no NH_4Cl); red, NH_4Cl added at 188 s; green, uncoupler added after 250 s. The actinic light intensity was 500 µmol $m^{-2}s^{-1}$. **Samples were dark adapted for 30 min before measurements.** Care was taken to ensure sample mixing throughout the whole procedure. For further information, see materials and methods. **Panel B**. NPQ, $(F_m\text{-}F_m')/F_m$, calculated from the relative fluorescence traces in panel A.

>2BHW:A|PDBID|CHAIN|SEQUENCE
RKSATTKKVASSGSPWYGPDRVKYLGPFSGESPSYLTGEFPGDYGWDTAGLSADPETFS
KNRELEVIHSRWAMLGALGSVFPELLSRNGVKFGEAVWFKAGSQIFSEGGLDYLGNPSL
VHAQSILAIWATQVILMGAVEGYRIAGGPLGEVVDPLYPGGSFDPLGLADDPEAFAELKV
KELKNGRLAMFSMFGFFVQAIVTGKGPLENLADHLADPVNNNAWSYATNFVPGK

>CveliaI_19753.t1| Fucoxanthin-chlorophyll a-c binding protein|
MKTVAATVCAFAAVSVDAFTLGGVKPVARSARSEMKMSFDDAPGSGKYGLPGFPIFNPF
DLSPEDEKFKEYRLKELKNGRLAMLGILGLAATELGARLPGNLNAGFDLPFKQDGPAFS
DIPGGFAAFPALPAAGWAQVALFVALMDQVFYKQTDADEVAPGITYGKPEDPEEYLDLR
NKELNNGRIAMIGLLAMTFQYYIGGATEFPYISK

Figure S2. **Sequence of LHCIIb and CLH used in the present study.** Sequence of LHCIIb (DB code: 2BHW, Standfuss et al. 2005) and CLH (CveliaI_19753.t1 taken from Tichý et al. 2013) polypeptides employed for *in silico* analysis.

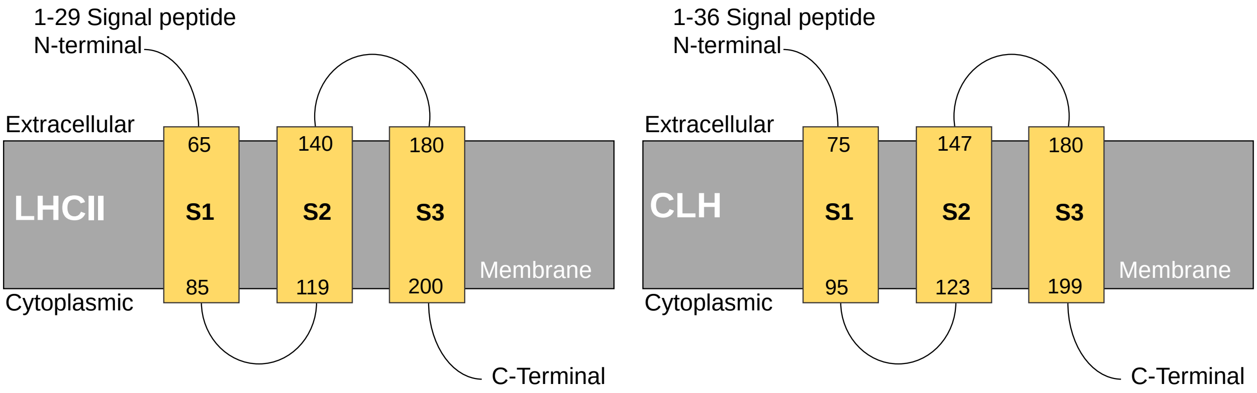

Figure S3. **Schematic overview of LHCII (left) and CLH (right) antenna protein structures used in the present study.** Structures were predicted using PHYRE 2 software. Transmembrane helixes are represented as yellow rectangles whilst stromal and luminal loops are shown as connecting arches. Residues located at the membrane edges are indicated.
